# Research protocol for a complex intervention to support hearing and vision function to improve the lives of people with dementia

**DOI:** 10.1186/s40814-017-0176-1

**Published:** 2017-09-11

**Authors:** Iracema Leroi, Annie Pye, Christopher J. Armitage, Anna Pavlina Charalambous, Fofi Constantinidou, Catherine Helmer, Ines Himmelsbach, Sarah Marié, Jahanara Miah, Suzanne Parsons, Jemma Regan, Chryssoula Thodi, Lucas Wolski, Abebaw Mengistu Yohannes, Piers Dawes

**Affiliations:** 1 Division of Neuroscience and Experimental Psychology, University of Manchester, Jean McFarlane Building, Oxford Road, Manchester, M13 9PL UK; 2 Manchester Centre for Health Psychology, School of Psychological Sciences, Manchester Academic Health Science Centre, University of Manchester, Manchester, UK; 3 Department of Health Sciences, European University Cyprus, Nicosia, Cyprus; 4 Centre for Applied Neuroscience, University of Cyprus, Nicosia, Cyprus; 5 INSERM, U1219 (Bordeaux Population Health), Clinical Investigation Center-Clinical Epidemiology, University of Bordeaux, 1401 Bordeaux, France; 6 Institute of Applied Research, Development and Continuing Education, Catholic University of Applied Sciences, Freiburg, Germany; 7 Research and Development Department, Essilor International, Paris, France; 8 Manchester Academic Health Science Centre, Central Manchester University Hospitals NHS Foundation Trust and the University of Manchester, Manchester, UK; 9 Department of Health Professions, Manchester Metropolitan University, Manchester, UK

**Keywords:** Dementia, Hearing impairment, Vision impairment, Complex intervention, Intervention mapping

## Abstract

**Background:**

Hearing and vision impairments are among the most common and disabling comorbidities in people living with dementia. Intervening to improve sensory function could be a means by which the lives of people living with dementia may be improved. However, very few studies have tried to ameliorate outcomes in dementia by improving sensory function. This paper describes the multi-step development of a new intervention designed to support hearing and vision function in people living with dementia in their own homes. At the end of the development programme, it is anticipated that a ‘sensory support’ package will be ready for testing in a full scale randomised controlled trial.

**Methods:**

This programme is based on the process of ‘intervention mapping’ and comprises four integrated steps, designed to address the following: (1) scoping the gaps in understanding, awareness and service provision for the hearing and/or vision impairment care needs of people with dementia using a systematic literature review and Expert Reference Group; (2) investigating the support care needs through a literature search, stakeholder surveys, focus groups, semi-structured interviews and an Expert Reference Group, leading to a prototype sensory support package; (3) refining the prototype by additional input from stakeholders using focus groups and semi-structured interviews; and (4) field testing the draft intervention using an open-labelled, non-randomised feasibility study, integrating feedback from people with dementia and their significant others to develop the final intervention ready for full scale definitive trialling. Input from the ‘patient and public voice’ is a cornerstone of the work and will interlink with each step of the development process. The programme will take place in study centres in Manchester, Nicosia and Bordeaux.

**Discussion:**

Quantitative and qualitative data analyses will be employed, dependent upon the sub-studies in question. Data from the steps will be integrated with consideration given to weighting of evidence for each step of the programme. This programme represents the logical development of a complex intervention to fulfil an unmet need. It is based on a theoretical framework and will lead to a subsequent full scale efficacy trial. The challenges in integrating the data and addressing the contextual issues across study sites will be scrutinised.

## Background

The purpose of this paper is to outline the steps we will take to develop a new intervention to support people with dementia and concurrent hearing and vision impairment living at home. The present research programme will shape the new intervention and its delivery structures, as *per* guidelines for the development of complex interventions [1] and the process of ‘intervention mapping’ [2].

### Hearing and vision impairment in dementia represents an unmet need

Hearing, vision and cognitive impairment are all within the top ten highest disease burdens in the European Union (EU) in terms of reduced quality of life and increased healthcare utilisation [3]. As people age, the incidence of developing one or more of these impairments rises dramatically, to the point whereby seven in ten Europeans over the age of 65 suffer either sight or hearing loss. Dementia and cognitive impairment steadily rise in prevalence in this same age group to the point where almost one third of Europeans at the age of 90 is affected [4]. Thus, the overlap between sensory and cognitive impairment is substantial and all three impact significantly on each other, resulting in a crucible of ‘multi-morbidity’.

Recent evidence has found that older adults experiencing comorbid cognitive and sensory impairment have greater difficulties in several aspects of their lives compared to individuals with dementia but without sensory impairments [5]. For example, people with comorbid cognitive and sensory problems may have more difficulty in locating themselves using visual or auditory cues and experience higher levels of disorientation and distress, which can lead to agitation, aggression and an increased prevalence of hallucinations and delusions [6]. In addition, such individuals are often more isolated from family interactions, participate less in social activities and hobbies, and are even more marginalised within the community, compared with those who have single morbidity or experience good cognitive or sensory health [7–11]. Such social isolation and disconnection can lead to depression and a more rapid overall decline in function [12]. Furthermore, caregiver burnout and physical exhaustion are amplified due to greater dependency for self-care and other activities of daily living and communication barriers [7]. In spite of this, the gaps and fragmentation in understanding, appropriate assessment, service provision and public awareness are significant across the EU. There is a marked deficit in appropriate detection and management of hearing and visual impairment in up to 94% of people with dementia [13–15]. This results in lost opportunities to foster mental well-being in elderly people [16, 17].

### Meeting the unmet need

In 2014, the European Commission’s Horizon 2020 programme announced a funding call (PHC-22) for research programmes with the aim of ‘promoting mental well-being in elderly Europeans’. Considering the significant negative impact of comorbid cognitive and sensory impairment on the mental well-being of elderly Europeans, addressing this unmet need is an important approach to answering the call. However, straightforward correction of hearing and vision impairments, by means of fitting hearing aids and lenses, is unlikely to succeed in the context of more serious cognitive deficits such as dementia. This is due to the added complexity of concurrent deficits as well as the low rate of access to vision and hearing services in elderly people [8, 18, 19]. Clearly, a more comprehensive approach to improving outcomes in people with dementia and sensory impairment is needed.

A possible solution to this identified need is to provide added ‘sensory support’ to people with dementia who live at home. The objective would be to enhance identification of impairment, improve uptake and adherence of corrective devices and assist in enabling this population to better manage daily life in the context of multiple deficits. It is hoped that through the provision of additional support, the benefits of correcting sensory impairments will be maximised. The ultimate aim of such an enhanced intervention is to improve ‘mental well-being’, which has been defined by the World Health Organization (WHO) as ‘a state of well-being in which the individual realises his or her own abilities, can cope with the normal stresses of life, can work productively and fruitfully, and is able to make a contribution to his or her community’(p10 [20]). ‘Mental well-being’ is also encompassed in the wider definition of health, which states that ‘health is a state of complete physical, mental and social well-being and not merely the absence of disease or infirmity’ (p3 [21]).

### Study protocol

#### Overall aims of the research programme

- To understand from different perspectives, the range of support care needs for people with dementia and concurrent hearing and vision impairment and the impact that these impairments have on outcomes such as quality of life and caregiver burden.
- To develop and field test a complex support care intervention that is theoretically informed and ready to be tested in a subsequent full scale definitive randomised controlled trial (RCT), should the outcomes of this development programme appear positive.

### Primary specific objectives

Step 1: Defining the gap

- To scope the gaps in understanding, awareness and service provision for the support care needs of people with dementia and hearing and/or vision impairment using stakeholder input and evidence from the literature;

Step 2: Developing a prototype intervention

- To investigate the specific support care needs required by the population in question using a mixed methods approach;
- To construct a prototype sensory support care package using data integration of the scoping and mixed methods investigations;

Step 3: Refining the prototype intervention

- To refine the prototype intervention into a draft intervention ready for field testing;

Step 4: Field testing the draft intervention

- To test the acceptability and feasibility of the draft intervention ready for full scale trialling;
- To ascertain the most appropriate outcome measures to be used in the full trial, including the impact on mental well-being, quality of life, caregiver burden, social connectedness, cognitive functioning and health economic measures.

### Secondary specific objectives

- To explore the experiences of people with dementia and hearing and/or vision impairment and those of their significant others.

## Methods/design

Due to the complexity of the intervention and the multiple contexts in which the research will take place, our approach to its development has closely followed the steps outlined by the process of intervention mapping [2]. In order to follow the logical and sequential steps in this process, a mixed methods study design is the most suitable approach. However, in evaluating each applicable ‘dimension of complexity’ (see Table 1), as outlined by the UK’s Medical Research Council (MRC) Guidance on the topic [1], we decided that a modified version of the mapping process was most suited to our needs. Thus, we adopted a non-linear structure to the research programme with both sequential and simultaneous sub-studies using quantitative and qualitative approaches [22].

**Table 1:** Description of ‘dimensions of complexity’ in the proposed intervention study as per Medical Research Council Guidance [1]

Dimension	Reason for complexity
Number of and interactions between components within the experimental interventions	Since we anticipate that no two individuals will have the same impairments, the assessment and management of impairment versus functional need will be undertaken by different individuals due to differing skill sets. For example, vision and hearing impairment requiring devices (glasses and hearing aids) will be undertaken by specialist clinicians (optometrists, ophthalmologists and audiologists), whereas the functional ability and environmental context, caregiver assessment and the resulting management strategy will be undertaken by a sensory support worker
Number and difficulty of behaviours required by those delivering or receiving the intervention	Whereas the clinicians will assess and correct hearing and vision impairments according to their standard good practice guidelines, the sensory support worker will undertake newly learned protocols, and will have to choose from a variety of elements, likely within different modules including: vision training, auditory training, caregiver education and training, information delivery, sign-posting and environmental assessment and correction
Number and variability of outcomes	In order to fulfil the remit of improving ‘mental well-being’ in elderly EU citizens, various outcomes need to be captured, including health-related quality of life (QoL), improved functional ability, social connectivity, caregiver factors, attainment of personal goals, as well as more easily quantifiable factors such as cognitive performance, level of depression and other behavioural disturbances.
Degree of flexibility or tailoring of the intervention permitted Implications for development and evaluation	Since no two individuals will have the same degree of cognitive and sensory impairment and functional ability, the intervention will have to be highly tailored and individualised, albeit within the structure of a reproducible, manualised and modular approach in which each person with dementia: caregiver dyad will be offered each module of the intervention package
A good theoretical understanding is needed of how the intervention causes change, so that weak links in the causal chain can be identified and strengthened	The background literature of existing evidence of potential mechanisms, as well as clinical experience, has suggested that each aspect of the intervention can be linked to identifiable intermediate impacts and final outcomes, and can be outlined in a logic model [24]

**Fig. 1:**
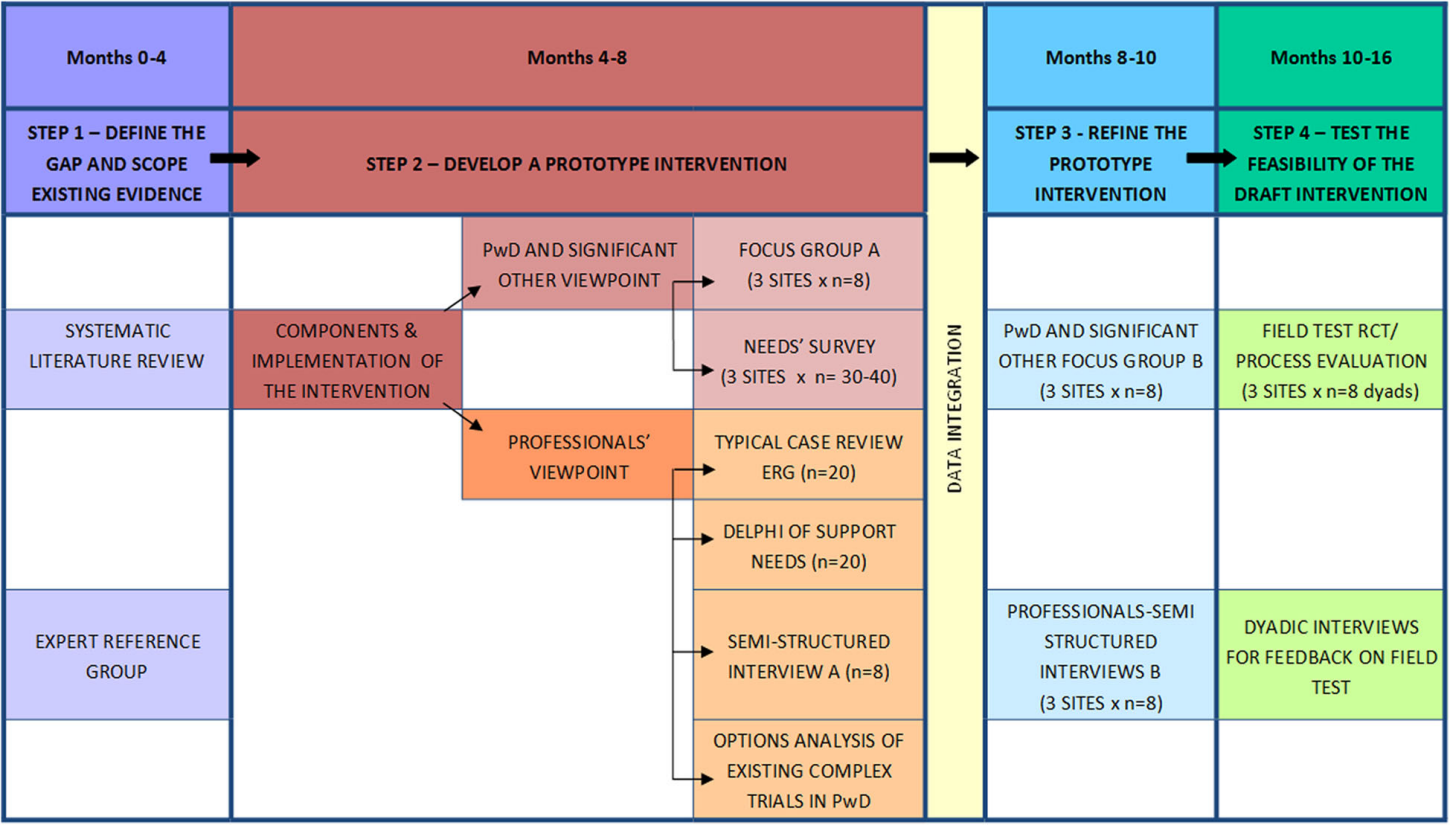
Flowchart of the sequential and simultaneous steps in the research programme. Person with dementia is abbreviated as PwD

Importantly, we carefully considered the different weighting of the evidence to be gleaned from each step in the development programme. Thus, in view of the crucial importance of understanding local EU contexts, as well as the paucity of published evidence on which to base quantitative methods, we have chosen to place a greater emphasis on exploratory and qualitative methods, over quantitative approaches. The steps comprising the full research programme are outlined in Fig. 1. The sub-studies included in each of the steps are outlined in Table 1.

Another important consideration for any intervention, particularly involving behavioural change, is that a theoretical framework be established prior to any development work. The principal aim of the intervention is to improve functional ability and quality of life by supporting sensory function. However, this can only be achieved by changing the behaviour of people with dementia and sensory loss to ensure that they are ‘living better’ as manifested by mental well-being and reduced caregiver burden. We have therefore chosen a comprehensive evidenced-based behavioural model around which the components of the SENSE-Cog intervention will be compiled, the Behaviour Change Wheel [23]. One of the aspects of the Behaviour Change Wheel is the COM-B component, which suggests that the key areas to be addressed to effect behavioural change (‘B’) include *capability* (‘C’), or the individual’s psychological and physical capacity to engage in the activity concerned; *opportunity* (‘O’) or all the factors that lie outside the individual that make the living better with dementia possible; and *motivation* (‘M’), or all those ‘brain processes that energize and direct behaviour, as well as goals and conscious decision-making’ (p5 [23]).

With the Behaviour Change Wheel as a framework, the likely structure of the final intervention will include various components including (1) identifying and correcting any vision or hearing impairment; (2) supporting adherence to the impairment correction with on-going advice and fittings; (3) identifying vision- and hearing-related functional deficits, facilitators and barriers; (4) addressing any identified skill deficit with vision and hearing training for the affected person and their significant other; (5) addressing any identified knowledge deficit with information provision and sign-posting; and (6) improving vision and hearing related functional deficits through home-based environmental aids. The intention is that the sensory support intervention will be highly individualised, be delivered at home, and involve the input of a caregiver or significant other. The overall aim of the intervention will be broad-based and targeted towards improving quality of life, reducing functional disability and increasing social connectedness. Finally, a cornerstone of the entire research programme is the input of patient and caregiver stakeholders by means of interlinking at each step with our ‘Patient and Public Voice’ (PPV) team.

### Aspects of intervention complexity to consider in designing the research programme

Our early scoping work in the literature and ‘gap analysis’ in our Expert Reference Group (ERG) identified several dimensions of complexity which need to be addressed in the development of the intervention. These are outlined in Table 2:

**Table 2:** Sub-studies included in the process of developing a complex intervention

Sub-study	Aim	Participants	Design	Setting	Data analysis
Expert Reference Group	To scope expert opinions on gaps in understanding of the support care needs and potential solutions for people with cognitive and sensory problems	Expert professionals from several disciplines (audiology, vision science, ophthalmology, optometry, health psychology, social work, occupational psychology, occupational therapy and social gerontology)	Three components: 1. Discussion of assessment and management of prototypical cases 2. Analysis of implementation of existing complex dementia trials 3. A modified Delphi to establish a list of support care needs	A two-day meeting in Athens, Greece	Prototypical case discussions will be transcribed and subject to content analysis
Focus group A	To scope the needs of people with dementia and sensory impairment	Three focus groups (*n* = 8 per group) will be conducted with people with dementia and sensory impairment, their study partners and professionals in three sites	Focus groups will be used to discuss support care needs of this population	Three focus groups will take place in each of three sites (total of nine groups): Manchester, Nicosia and Bordeaux	Conversations will be transcribed using MAXQDA software and subjected to content analysis. Cross-site differences will be explored within the analysis of these discussions
Needs analysis survey	To scope the support care needs of people with dementia and sensory impairment	People with dementia and sensory impairment and their study partner. Three sites will recruit between 30 and 40 dyads each	A series of questionnaires will be used to scope the needs, quality of life, degree of impairment, caregiver burden, mental and physical health of participants	Manchester will be the site for 40 participant dyads; Nicosia and Bordeaux will each recruit 30 dyads (total of 100 dyads).	Regression analyses will be used to explore the relationships between variables
Focus group B	To receive feedback on a draft intervention	Three focus groups (*n* = 8 in each group) will be run using professionals, people with dementia, and their study partners	Information from the preceding stages will be integrated to inform a draft intervention, which which will be presented to participants for comment and feedback	These will be the same three sites that participated in focus group A; however, the participants may differ between the two focus groups	As with focus group A, discussions will be transcribed and analysed using content analysis
Field Trial of draft intervention	To trial a ‘sensory support’ intervention for people with dementia and sensory impairment	Eight participant dyads (people with dementia and sensory impairment and their study partners) in each of three sites	A 12-week ‘sensory support’ intervention involving correction of hearing/vision impairments, communication training, ‘sensory proofing’ the home environment, and signposting to relevant services. Baseline and follow-up data will be collected along with process measures and reflective diaries	Three sites will recruit a total of 24 participant dyads: Manchester, Nicosia, and Bordeaux	Data from the diaries and verbatim data from the interviews will be analysed using a summative content analysis. Quantitative parametric data will be analysed using two-tailed *t* tests, with 95% CIs. Effect sizes using the Hedges’ g formula (similar to Cohen’s d), appropriate for small sample sizes will be calculated. Due to the small sample, only a large *p* value, 0.2, will be an indication of effect. The score distributions will be examined for degree of variability and ceiling and floor effects. Participants’ subjective report of the overall helpfulness of the intervention will give an initial impression of efficacy. An initial impression of treatment efficacy will be obtained by examining before and after-intervention measures of a variety of rating scales of dementia-related outcomes

At the end of this part of the research programme, we will have determined the following:

- What method we will use to assess participants’ (a) level of hearing and vision impairment and (b) range of functional needs in a home-based setting, in order to tailor the intervention to their specific needs.
- What the individual components (e.g. modules) of intervention will be.
- How the intervention will be implemented (e.g. duration, frequency and delivery of each component).
- What causal assumptions about mechanisms of producing change in a home-based setting will be made.
- How the intervention will work in the context of different EU settings.

### Participants

Throughout the programme, we will engage with four groups of participants in each of the three participating centres. The specific inclusion/exclusion criteria for each of these groups will be outlined in more detail in the description of each of the steps. All participants will be required to have the capacity to consent in order to participate. Capacity checks will be made in accordance with the UK’s Mental Capacity Act 2005 [24]. The four groups of participants are as follows:

i. People ≥ 60 years of age with mild to moderate dementia (due to Alzheimer disease (AD), vascular dementia or mixed AD/vascular dementia) and vision or hearing impairments. Individuals with severe dementia will be excluded from the study on the grounds that the field trial, which is the end point study in the development process, involves completing several outcome measures and engaging with a complex, multifaceted intervention, which may potentially place too much burden on individuals with more severe dementia.
ii. Study partners of the affected people, who will likely be spouses or unpaid caregivers (significant others).
iii. Expert professionals representing different disciplines pertaining to the programme’s goals, including geriatric psychiatry, audiology, vision science, ophthalmology, social gerontology, optometry, occupational therapy, social work, health psychology and statistics.
iv. Representatives of PPV who are part of the team’s Research User Group (RUG).

### Settings

The ERG will take place in Athens, an EU city with easy access for the experts and in close proximity to the SENSE-Cog consortium members from Greece who can support the operations of the group. The PPV consultations will take place in each of three clinical sites: Manchester, Nice and Nicosia. The clinical sub-studies (Needs Survey, Focus Groups and Field Test) will take place in each of three sites with the following sponsors: the University of Manchester, European University Cyprus, and the University of Bordeaux.

### Recruitment

Recruitment of expert participants will be informed by the literature and through professional contacts of members of the wider SENSE-Cog team, which is a multi-disciplinary consortium of professionals addressing all aspects of the study programme. Recruitment of the PPV members will take place in each of the relevant sites by placing notices in the newsletters and websites of local charities, support groups and existing PPV networks. Participants who are people with dementia and their caregivers or significant others will be recruited from memory clinics in Manchester, Bordeaux and Nicosia through direct contact with their treating clinician.

### Study design, methods and analyses for each step in the programme

In order to achieve our aims, we have divided the development programme into four distinct steps: (1) defining the gap in care and scoping the existing evidence of the impact of sensory deficits in dementia, (2) developing a prototype intervention, (3) refining the prototype into a draft intervention, and (4) field testing the draft intervention.

### Step 1: Defining the gap in care and scoping the existing evidence of the impact of sensory deficits in dementia

#### Systematic and rapid literature review

*Objective:* To assess the impact of treating hearing and vision impairment on (1) cognition, (2) rate of decline, (3) psychiatric symptoms, (4) hearing/vision-related quality of life, (5) health-related quality of life, and (6) caregiver burden for people with dementia.

*Method:* Electronic databases will be systematically searched using the key terms and their synonyms: dementia AND (hearing impairment/deaf OR sight/vision impairment) AND, (intervention OR rehabilitation OR management OR treatment OR outcome). Databases searched were Google Scholar, MEDLINE, Cochrane Central Register of Controlled Trials, EMBASE, PsychINFO, CINAHL, AgeInfo, Web of Science, Scopus, ComDisDome, Open Grey, ClinicalTrials.gov and the WHO international clinical trials registry. The database searches will be supplemented by searching through bibliographies of papers that match the eligibility criteria and via consultation with a network of health professional experts to identify additional grey literature that has not been found via the database search.

*Analysis*: Screening of both titles and abstracts will be done by two independent reviewers. All disagreements will be resolved by discussion or consultation with a third reviewer if necessary. Risk of bias assessment and data analysis will be completed and reported in a narrative review.

#### Gaps identified from professionals’ expertise and experience

*Objective:* To ascertain the views from professionals within the domains of dementia, hearing and vision impairment regarding the ‘gaps and solutions’ in the support care needs of people with concurrent problems.

*Method*: Face-to-face guided discussion and written survey in the context of an ERG, held over 2 days in Athens, Greece. Each participant will be asked to present their pre-prepared answers to the following questions prior to the meeting: ‘What are the key gaps in knowledge in the field from your professional perspective?’; ‘What problems might you encounter if the person has concurrent hearing/vision/cognitive problems in a clinical setting?’; and ‘What solutions might you offer to the problems that might arise?’.

Directions will be given to the experts advising them that the focus will be on their direct clinical experience and research expertise in the field.

*Participants*: Expert professionals (*n* = 20), identified through the rapid literature review and personal contacts of the members of the SENSE-Cog consortium, will be invited to an ERG. The experts represent the disciplines described above and will come from member countries of the SENSE-Cog consortium (England, France, Germany, Cyprus, Greece) in addition to Canada and Wales.

*Analysis*: We will use qualitative thematic analysis of responses to describe the range and content of the expert opinions.

### Step 2: Developing a prototype intervention

In this step, the overall objective is to compile a battery of intervention components (e.g. modules) to support hearing and vision function in the home setting, as well as examples of the implementation of complex interventions in dementia, to form the basis of a prototype intervention. To do this, we will undertake several study activities to determine the specific support care needs and method of implementation from the perspective of the expert professionals as well as those people with dementia and their caregivers or significant others. We will also determine which type of tool or assessment will best ascertain the level of impairment and functional ability of the affected person in order to tailor the intervention specifically to their needs.

#### Identifying the components and implementation of the intervention from the affected person and caregivers or significant others’ viewpoint

*Objective:* To ascertain the support care needs of people with dementia and concurrent sensory impairment from the perspective of those affected and their caregivers or significant others.

*Method:* This will be a two-part study in each of the three clinical sites: Manchester, Nicosia, and Bordeaux. The first part will be a researcher-administered survey in people with dementia with sensory impairment living at home, and their caregiver (study ‘dyads’). The survey will consist of three sets of questionnaires: (1) for the people with dementia; (2) for the caregiver or significant others (‘study partners’); and (3) for the dyad, to elicit a consensus response. The questionnaires are outlined in Table 3.

**Table 3:** Questionnaires to be administered to affected participants, significant others and for consensus response in the survey component of the prototype intervention development

Affected participant	Significant other
Socio-demographic information	Socio-demographic information
Hearing Handicap Inventory for Elderly Screening (HHIE-S) [25–27]	Geriatric Depression Scale short form (GDS-s) [28]
Veterans Affairs Low Vision Visual Functioning Questionnaire (LV VFQ-20) [29]	Short version of the Burden Scale for Family Caregivers (BSFC-S) [30]
Six-item De Jong Gierveld Loneliness Scale [31]	Six-item De Jong Gierveld Loneliness Scale [31]
Geriatric Depression Scale short form (GDS-s) [28]	Short version of the Information Questionnaire on Cognitive Decline in the Elderly (IQ CODE) [32]
DEMQOL-health related quality of life in people with dementia [33]	
Six-item Cognitive Impairment Test (6CIT) [34]	Patient Health Questionnaire-15 [35]
Final scale from the Clinical Dementia Rating (CDR) [36]	
Supportive Care Needs Survey (adapted for this population with permission from the authors) [37]	

The second part of the study will consist of a set of focus groups or semi-structured interviews (focus group A) with people with dementia and their caregivers. Focus group ‘A’ will attempt to ascertain the support care needs of individuals living with dementia and sensory impairment.

*Sample size*: For the needs analysis survey, each of the three clinical sites will recruit 30 to 40 dyads (*n* = 100 dyads in total).

There will be eight individuals recruited into each focus group. For any individuals who are unable to attend focus groups, home-based semi-structured interviews using the same schedule of questions will be offered.

*Participants:* The needs analysis survey and focus groups will recruit both people with dementia (individuals with a diagnosis of dementia and sensory impairment) and their caregivers.

*Analysis:* Survey data will be analysed using descriptive and quantitative methods and regression analyses in attempt to determine which factors contribute to support care needs and the relationships between these factors. Focus groups will be audio recorded, transcribed and subjected to content analysis [38]. Based on the literature and theoretical background, a research question will be developed to structure the identification of relevant data. The next aspect of data coding will involve deriving categories from the data that has been identified as being relevant to the research question. The analysis will explore the major categories and discussion points, in order to identify key areas of need for this population. The intervention will then be developed with the objective of targeting areas in which the need for help is greatest.

#### Identifying the components and implementation of the intervention from the professionals’ viewpoints

In order to elicit the viewpoints of expert professionals on the most suitable components of the intervention and how it should be implemented, we will use two methods: (i) the ERG (consisting of three components) and (ii) semi-structured interviews.

#### ERG

*Objective:* To elicit the opinions of expert professionals on the assessment and management of people with concurrent dementia, hearing and/or vision impairment.

*Method*: ERG with three activities: (a) guided discussion of the assessment and management of three prototypical cases, (b) analysis of existing complex dementia trials’ implementation methods, and (c) modified Delphi process of a list of support care needs.

- *Guided discussion of prototypic cases*: Here, we will ask clinical professionals from each of the three domains (dementia, hearing and vision impairment) to contribute to a clinical scenario reflecting typical cases of concurrent impairment. For each of the three cases, the ERG will be divided into two working groups, balanced for profession, gender, country of practice and academic versus clinical activity, which will be randomly changed for each new case discussion. Scribes will take detailed minutes of the discussions which will be used as data material. They will be analysed via content analyses using MAXQDA software [39]. The protocols of scribes will be subjected to the question of which gaps and solutions the experts reported. By coding thematically, comparisons between the three groups will inform the further development of the intervention protocols.
- *Analysis of implementation methods of existing trials of complex psychosocial interventions in people with dementia*: Here, we will use the forum of the ERG and invite expert opinions on the implementation of four recently published protocols for complex psychosocial interventions in people with dementia with intact or corrected sensory function. Each of these trials was chosen by the study team due to the robust methodology involved (randomised, placebo controlled, rater-blinded, fully powered efficacy trials), the target study population (dementia in the mid to moderate range, including a range of diagnoses), home-based setting, and the adherence to the MRC Guidance on complex interventions [1]. These protocols were ‘GREAT’, trialling cognitive rehabilitation therapy in dementia [40]; ‘iCST’, trialling individualised cognitive stimulation in dementia [41]; and ‘ATTILA’, trialling assisted technology in dementia [42]. Each of these trials uses different implementation methods, including a structured manualised approach with a set number of sessions (iCST), a semi-structured approach with individually established goals and a set number of sessions (GREAT), and a highly pragmatic trial with interventions varying according to the local service provider (ATTILA). Outcomes in all these trials included quality of life, functional, cognitive, behavioural, health economic and caregiver outcomes. We will also analyse an additional trial involving a non-randomised complex intervention for older people with vision impairment but without dementia, the LOTSE trial [43]. LOTSE utilises an individualised approach for persons with late-onset vision loss. It is composed of ten standardised intervention modules (on psychosocial issues, information and practical help). After a thorough first interview with the patient, a social worker decides which of these modules are the most appropriate for the persons to address. With this stepwise process which is flexible enough to change the focus throughout consecutive meetings, vision specific topics are always addressed concerning the actual needs of the patient and thus accommodate the progressive status of age-related vision loss and possible multi-morbidity. Outcomes in LOTSE address quality of life, functional ability and well-being.
  For each of these studies, after the study outline and implementation procedures are detailed, the ERG will be invited to appraise the options with a view to adopting the most suitable method for delivering our sensory support intervention. The options’ appraisal will use the ‘TOWS method’ [44]. Briefly, this is a decision-making tool in which a matrix of external threats (‘T’) and opportunities (‘O’) based on the different methods used in the trials will be mapped against the internal strengths (‘S’) and weaknesses (‘W’) of what the study team can deliver. The outcome of this decision tool will guide the implementation model for the future sensory support trial.
- *Modified Delphi process:* For this activity, we will again call on the ERG to provide input in order to derive a hierarchical list of support care needs for the participant population. The Delphi process will consist of three phases. *Phase I: preparation of initial item list*: This phase was completed prior to writing this protocol. An initial rapid scoping of the literature identified a care preference list as part of a survey for a different disease area. This list, being comprehensive and pragmatic, was modified in an iterative and sequential fashion by nine professional experts across the key disciplines involved in order to inform the content of a potential support care checklist for people with dementia and hearing and/or vision impairment. This modified list was presented for comment and modification to patient and public contributors consisting of stakeholder participants (affected people and their significant others). Items from this version will be used in phase 2 of the professional expert consensus process. *Phase 2*: Experts will be asked to rank in preferential order the relative importance of the items of support care preferences from the list derived in phase 1. These are clustered in four domains: hearing, vision, cognition, and hearing, vision and cognition combined. Each domain contains up to nine items and will be ranked by the experts according to each of the following categories: cost, availability, tolerance or acceptability for the person with dementia and their significant other, perceived efficacy in improving patient quality of life, the degree of skill required by the therapist and adaptability across cultures and languages and different EU health settings. The initial ranking will take place at the end of the 2-day ERG meeting in Athens in which the relevant issues will have been discussed by the ERG in various group activities. The outcome of the first ranking will then be examined for frequency of responses in the various domains and categories and, if necessary, a subsequent set of care preferences will be drawn up for ranking by the group at a later date in phase 3 to arrive at a refined list of support care preferences to inform the intervention.

#### Semi-structured interviews with professionals

*Objective:* To ascertain the support need of people with dementia and concurrent sensory impairment from the perspective of professionals.

*Method:* Semi-structured interviews lasting 1–2 h will take place with a range of relevant professionals, both academic and clinical, to determine the challenges faced by individuals living with concurrent sensory and cognitive impairment. Interviews will follow a similar structure to the focus groups carried out with the people with dementia and their caregivers. The discussions will be carried out using the guidelines of focus group ‘A’, concerning the support care needs of individuals living with dementia and sensory impairment.

*Participants:* A series of different professionals (*n* = 8 per study site) will be approached for interviews including optometrists, audiologists, clinical psychologists, community psychiatric nurses, occupational therapists and geriatric psychiatrists.

*Analysis:* Interviews will be transcribed and compiled with the data from other participating sites using MAXQDA software [39]. Following this, all data will be subjected to a qualitative content analysis addressing questions on life situations of people with dementia and caregivers from the perspective of professionals, using the COM-B model for deductive needs analysis and structuring the ideas on intervention targets and challenges.

#### Determining the method of assessment of impairment and level of functional ability

*Objective*: To ascertain the best method or tool to assess the person with dementia’s level of sensory impairment and functional needs in a home-based setting in order to tailor the intervention to their specific impairment correction and support care needs.

*Method*: Through a discussion and consensus process within the study steering group, we will extract this information from three sources: (1) the systematic literature review above, (2) the cumulative expertise and experience of the professionals’ attending the ERG through the ‘gaps and solutions’ survey above, and (3) the guided discussion of prototype cases mentioned also undertaken during the ERG.

### Step 3: Refining the prototype into a draft intervention

In this step, components of a prototype intervention developed from the intelligence gathered in steps 1 and 2 will be refined through input using qualitative methods. This will include semi-structured interviews with a range of relevant professionals, semi-directed focus groups (focus group B) with the person with dementia/significant other dyads, and an ERG using focus group methodology with a selected group of specific professionals, occupational therapists. For each study activity, the components of a prototype intervention will be presented and semi-structured questions posed in order to elicit views and opinions regarding the utility, feasibility, acceptability and tolerability of each component of the prototype intervention.

#### Semi-structured interviews and focus group ‘B’

*Objective:* To determine the methods of implementation and the components of the prototype intervention from the viewpoint of people with dementia, their caregivers, occupational therapists and relevant professionals.

*Method:* Focus groups lasting approximately half a day will be run for each of the three groups of participants; people with dementia, caregivers and occupational therapists. Semi-structured interviews will also take place with relevant professionals. The aim of these interviews and focus groups will be to review a prototype intervention, devised from the materials currently available and the intelligence outputted from steps 1 and 2. The materials will be evaluated with regard to their usefulness, their ease of implementation and their ability to improve well-being in people with dementia and their caregivers. Issues such as intervention inclusion/exclusion criteria, reach, participant acceptability and tolerance and long-term sustainability will also be discussed with the professionals.

*Participants:* Focus groups will consist of 5–8 participants per group (person with dementia, caregiver and occupational therapists) per site (Manchester, Bordeaux and Nicosia). Eight one to one, semi-structured interviews will take place with professionals.

*Analysis:* As with the other interview and focus group data, the conversations will be transcribed and subjected to content analysis [38].

### Step 4: Field testing the draft intervention

The final step in the programme will be to field test the draft sensory support intervention with a participant population representative of the group who will participate in the subsequent RCT of the intervention, should the results of the field test be positive.

*Objective:* The field study will have three objectives: (1) to undertake a process evaluation of the components and implementation of the intervention, including reach and fidelity issues, acceptability and tolerability of the intervention for participants as well as the contextual issues; (2) to examine the operational aspects of a future trial design, including recruitment and retention and training methods and materials; and (3) to explore the feasibility and face validity of potential outcome measures for the future trial. As the field trial comprises a complex, multi-faceted study, it is described elsewhere in detail (Regan J, et al. Improving hearing and vision in dementia: protocol for a field trial of a new intervention, in submission). The current description is comprised of a brief overview of the field trial in the context in which it has been developed.

*Method:* This will be an open-label feasibility study in people with dementia and sensory impairments and their caregivers in each of three clinical sites. There will be no control group. Participants, researchers and sensory support workers will not be masked to intervention assignment and all participants and their caregivers will receive either the full draft intervention package or certain components of the package decided a priori. Whether or not the individual receives the full intervention or a reduced version will depend on their availability, interest and need for support in certain areas.

*Participants:* The study population for the field trial will reflect the intended study population of the potential large-scale RCT as closely as possible and will include two groups of participants, all ≥ 60 years and living at home: (1) those with a diagnosis of dementia^1^ mild-to-moderate stage (Montreal Cognitive Assessment ≥ 12 [45]), and sensory impairment; and (2) their caregivers/significant others who will act as ‘study partners’. Hearing and vision function will be classified according to a priori established criteria of impairment. Caregivers or significant others will be included if they have a personal relationship with the participant, such as a family relation, friend or neighbour and can provide information about the participant’s condition and are willing to take part in the study themselves. Any participant unlikely to comply with follow-up (e.g. due to an unstable medical condition or having an urgent need for a sensory intervention) will not be included in the study.

*Sample size*: In this feasibility study, a formal power calculation will not be undertaken as no inferential analyses of efficacy are planned. Instead, we will enrol 24 participant:caregiver dyads across three clinical sites (8 dyads per site) which should give sufficient opportunity to extract the information needed from the study and fulfil the objectives.

*Setting:* The study will take place in clinic settings and participants’ own homes in each of the three study sites: Manchester, Nicosia and Bordeaux.

**Fig. 2:**
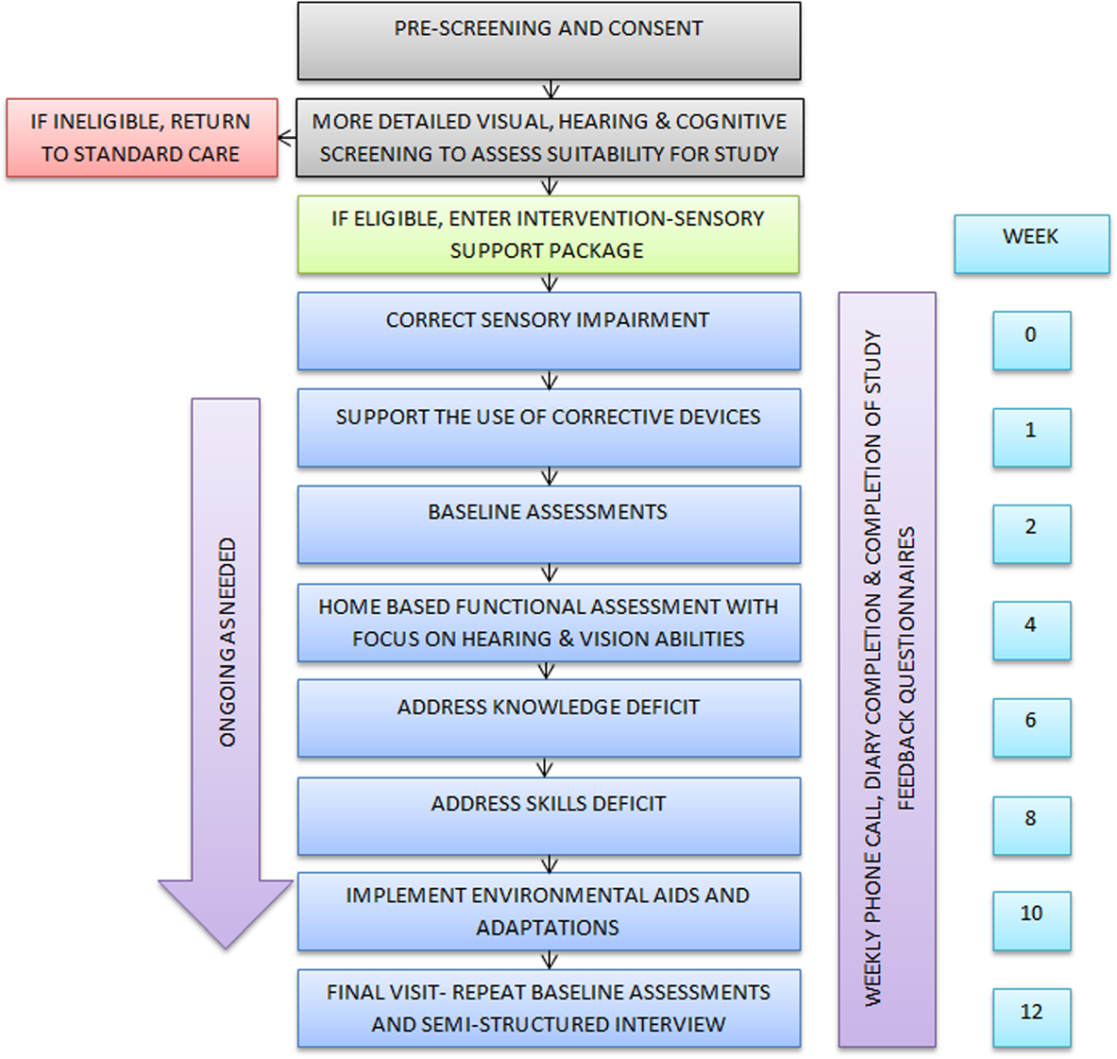
Flowchart of the components of the field test

*Study procedures:* The procedures are summarised in Fig. 2. Briefly, the study will take place over a period of three months with a series of interventions offered by professionals (opticians and audiologists), sensory support workers (SSW), and researchers undertaking the outcome assessments. Following recruitment, participants will be required to provide informed consent to participate. In order to overcome potential issues with consent presented by functional and sensory impairments, the researcher will be able to fill in the consent form on behalf of the individual and countersign to confirm that verbal consent has been provided. Following the signing of consent, potential participants will be screened for suitability to be included in the study. Screening will entail brief checks of hearing (using the SIEMENS HearCheck), vision (using the PEEK acuity smartphone application) and cognition (using the MoCA). For those who pass screening, a clinical assessment of vision and hearing status will be arranged. Vision status will include refraction, visual acuity, fields and contrast sensitivity. Hearing status will include otoscopy, tympanometry and pure tone audiometry. If surgical or invasive corrections are required (e.g. for cataracts or macular degeneration), the participant will be withdrawn from the study. If non-surgical sensory corrections or improvements can be provided with devices (hearing aids and/or lenses), these devices will be offered and fitted. Fitting and uptake of the new devices will be supported by the SSW who will visit the participant at home. Once impairments have been optimally corrected, a home-based assessment of vision and hearing functional ability will be undertaken in order to assess the specific support care needs of the participant and their caregiver. Based on the needs identified, a sensory support package will be offered to the dyad, based on the COM-B framework outlined above [23]. The specific components (e.g. content of each module) and manner of implementation (e.g. frequency and duration of support visits) will be determined from the intelligence gathered in steps 1 to 3 of the research programme. The final protocol outlining the study procedures and the draft intervention to be field tested will be prepared at the end of step 3. A researcher will undertake a series of process evaluations at each step of the protocol in order to ascertain such outcomes as feasibility, tolerability, acceptability and fidelity and reach of the intervention. The evaluations will involve participant diary keeping, direct researcher observations of visits, participant satisfaction scales, and SSW and researcher feedback. At the end of the intervention, each participant:caregiver dyad will participate in a 1–2 h semi-structured interview with the researcher to give feedback on the intervention components and implementation. A similar evaluation will be undertaken to ascertain successful aspects of trial procedures and outcome measures used. The outcomes to be explored will include measures of quality of life, mood, well-being, cognitive and functional ability, caregiver indices (e.g. burden, stress, well-being, and mood) and health economic measures.

*Analyses:* Demographic and baseline data will be fully described, and all outcome data will be analysed and reported. The quantitative evaluation will be a description of the rates of recruitment and retention over the period of the study, the tolerability and utility of the assessment procedures, as well as a description of the participant reaction to the intervention components and the frequency and duration of the therapy sessions as reflected by the in-house post-session rating scale. Fidelity of the intervention, as per Lichtstein et al. [46], will be analysed for content, as derived from the SSW, researcher and participant outcome data. The qualitative data from the semi-structured interviews will follow the same methods as described above for steps 2 and 3.

The outcome of the Field Trial will determine whether we will proceed to a full scale efficacy trial. Reasons for not proceeding to a full trial will be determined on the basis of the detailed process analysis of the draft intervention components and implementation, as well as the operations of the trial procedures being tested in the Field Trial. These will include measures of participant acceptability, tolerability, and feasibility of the intervention; the practical aspects of the logistics circuit regarding the supply and delivery of the corrective devices (glasses and hearing aids); the meaningfulness and feasibility of the outcome measures; and other key aspects informing a full scale trial. The specifics of these interventions are outlined in a protocol of the SENSE-Cog Field Trial (Regan J, et al. Improving hearing and vision in dementia: protocol for a field trial of a new intervention, in submission).

### Patient and public viewpoint contribution

Until recently, the involvement of older people with mental health problems, dementia and sensory impairments in research was minimal and considered impractical. This view is now changing, and it is recognised that only by fully including the views and opinions of older people with mental health problems and dementia and their caregivers will the findings from research have meaning, more effectively meet the needs of older people and enable the promotion of mental well-being. Thus, involvement of the ‘patient and public’ voice (PPV) will take place at every step of the programme, with the concepts of empowerment, self-management and participation acting as a guiding framework. The inclusion of the patient and public voice in this research is critical to ensure the quality, relevance, sensitivity and appropriateness of the final intervention that will be taken forward for the definitive efficacy trial.

*Objective:* To ensure that PPV fully informs each step of the research programme.

*Method*: In order to enable involvement of PPV, we are currently establishing three Research User Groups (RUGs) (in Manchester, Nicosia and Bordeaux), consisting of approximately five people with dementia and/or sensory impairments and/or their caregivers or significant others. Obtaining RUG feedback on each component of the intervention will be an iterative process, involving initial feedback from RUG members, making suggested modifications, and obtaining further feedback from a wider pool of RUG members. The PPV Facilitators for each RUG will be interviewed along with the study investigators to report on the RUG feedback and suggested changes to the intervention package. The iterative approach will act as a means of undertaking rapid tests of change to incrementally adapt the components and implementation of the prototype intervention. The points at which we will obtain the input and/or feedback are outlined in Table 4.

**Table 4:** Types of feedback required from ‘Patient and Public Voice’ in the development of the intervention

Type of feedback required	Method to obtain this
Study design and procedures	Consultation using structured interviews
Participant information sheets	Consultation using structured questionnaires
Intervention components	Plan, Do, Study, Act (PDSA) cycles [46]
Intervention implementation	PDSA cycles

*Analysis*: Semi-structured interviews will be transcribed and analysed using content analysis.

*Ethics:* In order to undertake the PPV reviews and use the feedback, public contributors will be actively involved and act as expert advisors, providing valuable knowledge and expertise based on their experience. This strand of work will be a review of research practice methods and will therefore not require ethical approval [47]. Since the data will be fully retained within the university’s network, there will no potential for any breach of RUG confidentiality.

### Integration of outputs from the development programme

The various outputs of the steps of the programme will be synthesised using guidance outlined in Brannen [48]. This will involve the integration of qualitative and quantitative data with consideration given to weighting of evidence. The methods used to do this include searching for corresponding lines of evidence to validate findings from different sources, elaborating and providing greater context and details to findings and using contradictory data to generate further research questions. The weighting of lines of evidence will be by agreement of the study steering group and informed by prior evidence available and the strength of the method of data collection.

### Data management and sharing

Research data from the various studies undertaken at each step of the programme and at all three clinical study sites will be collected and managed in Manchester. This will follow the procedure outlined in the SENSE-Cog Data Management Plan, which has already been approved by the European Commission through the Horizon 2020 programme. All identifiable and non-identifiable participant data will be stored separately in locked cabinets at the respective study sites. Restricted access will be limited to only those identified as investigators on the study and the relevant regulatory authorities. Participants will be assigned a unique identifier code on entry into the study which will be used on all subsequent paperwork and any electronic documents or audio files to enhance confidentiality. Both quantitative and qualitative data will be entered into pseudonymised databases held on the clinical sites’ computers. Audio and video recorded data will be destroyed immediately after written notes have been checked and supplemented. Records will be destroyed 10 years after the end of the study.

*Data transfer between sites*: All data transferred between sites will be fully anonymised prior to transfer. Transfer of electronic data will be done using encrypted files. The local site will retain a copy of any paper data and send the source files, via recorded delivery, to the University of Manchester for analysis.

### Dissemination

The dissemination plan for the programme will have two key objectives: (1) a set of internal reports of the outcomes of each activity of the programme in order to inform the study team of the next steps in the overall intervention development and (2) manuscripts and reports with outcomes of selected activities for external communication. These latter will comprise lay communications for the SENSE-Cog website and for public newsletters, as well as scientific manuscripts for publication in open-access peer reviewed professional journals. Specifically, the scientific outputs will include the systematic literature reviews, a support care needs’ analysis from the patient and significant other perspective, a support care needs’ analysis from the professionals’ perspective, a description of the final field tested intervention package, a process analysis of the field trial with a report of preliminary efficacy findings and the protocol of the development programme. The lay reports, led by the PPV groups, will be made available in each of the SENSE-Cog partner sites through local charity newsletters, websites and bulletins. The wider SENSE-Cog programme has a comprehensive dissemination strategy which will be utilised with regard to the output of the current work. This includes outputs posted on social media sites.

### Timeline

The overall timeline will take 16 months to complete. Months 0–4 will concentrate on step 1, months 4–8 will concentrate on step 2, months 8–10 will be focused on refining the prototype intervention and step 4 will be completed between months 10–16. The lengths of time spent on each of the steps of the development programme are outlined in Fig. 1.

## Discussion

One of the key challenges faced by this research programme is the relative lack of existing evidence to support the structure of a complex intervention for hearing and/or vision impairment in people with dementia. For this reason, reliance on the ERG as well as adapting existing approaches from other disease areas will be important. Another key challenge is that there are significant difficulties inherent in the evaluation of complex interventions such as ‘sensory support’. In particular, there will be the need to maintain standardisation across sites, while still capturing the important and interesting contextual differences of the sites, all of which have different levels of resources, health and social economies, cultures and languages [49].

Another critical consideration is the need to manage the organisational complexities of a potential future full scale RCT which, if it proceeds, will be rolled out to five sites, comprising the initial three sites of the development programme and additional sites in Athens and Nice. The field trial will be crucial to establish the cross-site methods of the future RCT and to develop and rehearse the standard operating procedures for each step in the protocol.

## Abbreviations

ERG: Expert Reference Group
EU: European Union
PDSA: Plan, Do, Study, Act
PPV: Patient and Public Voice
RCT: Randomised control trial
RUG: Research User Group
SSW: Sensory Support Worker
WHO: World Health Organization
